# An Interpretable Machine Learning Framework for Rare Disease: A Case Study to Stratify Infection Risk in Pediatric Leukemia

**DOI:** 10.3390/jcm13061788

**Published:** 2024-03-20

**Authors:** Irfan Al-Hussaini, Brandon White, Armon Varmeziar, Nidhi Mehra, Milagro Sanchez, Judy Lee, Nicholas P. DeGroote, Tamara P. Miller, Cassie S. Mitchell

**Affiliations:** 1 Laboratory for Pathology Dynamics, Georgia Institute of Technology and Emory University, Atlanta, GA 30332, USA; 2 Department of Electrical and Computer Engineering, Georgia Institute of Technology, Atlanta, GA 30332, USA; 3 Department of Biomedical Engineering, Georgia Institute of Technology and Emory University, Atlanta, GA 30332, USA; 4 Aflac Cancer and Blood Disorders Center, Children’s Healthcare of Atlanta, Atlanta, GA 30322, USAtamara.miller@emory.edu (T.P.M.); 5 Department of Pediatrics, Division of Pediatric Hematology/Oncology, Emory University, Atlanta, GA 30332, USA; 6 Machine Learning Center at Georgia Tech, Georgia Institute of Technology, Atlanta, GA 30332, USA

**Keywords:** pediatric leukemia, infection, artificial intelligence, machine learning, infection, natural language processing

## Abstract

**Background**: Datasets on rare diseases, like pediatric acute myeloid leukemia (AML) and acute lymphoblastic leukemia (ALL), have small sample sizes that hinder machine learning (ML). The objective was to develop an interpretable ML framework to elucidate actionable insights from small tabular rare disease datasets. **Methods**: The comprehensive framework employed optimized data imputation and sampling, supervised and unsupervised learning, and literature-based discovery (LBD). The framework was deployed to assess treatment-related infection in pediatric AML and ALL. **Results**: An interpretable decision tree classified the risk of infection as either “high risk” or “low risk” in pediatric ALL (*n* = 580) and AML (*n* = 132) with accuracy of ∼79%. Interpretable regression models predicted the discrete number of developed infections with a mean absolute error (MAE) of 2.26 for bacterial infections and an MAE of 1.29 for viral infections. Features that best explained the development of infection were the chemotherapy regimen, cancer cells in the central nervous system at initial diagnosis, chemotherapy course, leukemia type, Down syndrome, race, and National Cancer Institute risk classification. Finally, SemNet 2.0, an open-source LBD software that links relationships from 33+ million PubMed articles, identified additional features for the prediction of infection, like glucose, iron, neutropenia-reducing growth factors, and systemic lupus erythematosus (SLE). **Conclusions**: The developed ML framework enabled state-of-the-art, interpretable predictions using rare disease tabular datasets. ML model performance baselines were successfully produced to predict infection in pediatric AML and ALL.

## 1. Introduction

Acute lymphoblastic leukemia (ALL) and acute myeloid leukemia (AML) can occur at any age. While ALL and AML are among the most prevalent types of childhood acute leukemia, pediatric acute leukemias remain exceedingly rare. The incidence of AML in infants is 1.5 per 100,000 individuals per year, 0.9 per 100,000 individuals aged 1–4, and 0.4 per 100,000 individuals aged 5–9 years; after age 10, it gradually increases into adulthood, up to an incidence of 16.2 per 100,000 individuals aged over 65 years [1]. The rarity of pediatric ALL and pediatric AML means that research patient cohorts are composed of relatively small sample sizes, which has limited attempts to utilize cutting-edge machine learning (ML) techniques for pediatric leukemia clinical decision support. The ability to apply machine learning (ML) to standard available pediatric acute leukemia clinical data could provide a way forward in identifying opportunities for personalized therapeutic management.

Children with ALL or AML are treated with combination chemotherapy regimens. While effective in treating the leukemia, these regimens lead to periods of neutropenia and can cause significant treatment-related toxicities, including infection [2,3]. Prior studies show prolonged immune dysfunction in children for years after undergoing chemotherapy [4,5,6,7], which can make them particularly susceptible to infection. According to a recent study, infection is the most common cause of hospitalization in the first 3 years following treatment for pediatric leukemia [8]. Additionally, infection remains one of the most common causes of death in pediatric leukemia patients [9].

Prophylactic antibacterial or antifungal treatments are an option [10,11,12]. However, physicians must balance the prophylactic prevention of infection with the risk of anti-infectious drug resistance and polypharmacy. To this end, it would be prudent to classify patients into risk categories according to their likelihood of developing an infection. The goal would be to prescribe prophylactic anti-infectious agents to only the highest-risk group of patients who are most likely to develop a life-threatening infection.

ML presents an opportunity to potentially optimize the clinical management of pediatric acute leukemias. ML has rapidly become a cornerstone in medical research due to its ability to create complex models and analyze vast amounts of data [13,14]. In the context of leukemia, applications of ML have largely focused on adult leukemia cohorts, which have larger sample sizes than their rarer pediatric counterparts. For example, ML has been used to identify leukemia risk factors [15] and enhance diagnostic methods [16]. Classification models have been designed using blood counts [17] and blood transcriptomics [18] to identify cancer cell lines. ML has also shown promise in forecasting leukemia therapeutic outcomes using standard clinical data [19]. Additionally, ML has successfully predicted the likelihood of remission and drug sensitivity based on patient-specific gene expression data [20,21].

However, rare diseases, including pediatric leukemias, have a few attributes that make ML more challenging [22]. A recent review by Ramesh and colleagues outlined some of the challenges in applying artificial intelligence (AI) in rare pediatric cancers [23]. First, ML does not perform as well with small sizes. In particular, deep learning requires a very large number of observations. Second, most rare diseases have a large degree of variability in both patient features and patient outcomes [24]. The large variability is compounded by the smaller samples sizes. Third, most rare disease datasets are primarily composed of tabular data [25]. These datasets usually lack a plethora of rich features for the model to use as predictors. Fourth, models need to be interpretable in order to be trusted by clinicians to inform clinical decisions. Notably, interpretable models are sometimes referred to as “explainable AI” [26,27]. As such, there is a known trade-off between less interpretable black box methods, which may be more accurate, and more interpretable glass box methods, which may result in slightly lower performance accuracy. Here, “interpretability” is defined by the transparency of the underlying ML model decisions and especially how the model’s decisions can be explained by real-world domain expertise [28].

With these challenges in mind, a generalizable and interpretable ML framework was developed for small tabular datasets, which are common in rare disease research. The framework was applied to a rare pediatric disease tabular dataset to predict the development of infection in children undergoing treatment for AML or ALL. The presented ML framework paves the way for the improved research analysis of and clinical support models for rare disease. Specifically, the case study baselines provide an important foundation for future research focused on optimal infection prophylaxis for children with AML or ALL. The contributions of this work are as follows.

- A generalizable and interpretable ML framework was constructed to evaluate small, tabular clinical datasets. The primary incorporated modules were data preparation, supervised learning, unsupervised learning, and literature-based discovery. Each integrated method within each module was assessed and optimized to improve the accuracy, utility, and generalizability of the overall framework result(s).
- Pediatric AML and ALL patients were successfully stratified into high infection risk or low infection risk groups using supervised classification models.
- Supervised learning regression models predicted the discrete number of bacterial or fungal infections based on defined pediatric AML and ALL patient features.
- Unsupervised learning analyses determined which pediatric AML and ALL patient features and chemotherapy drug regimens explained the most variance in the development of infection.
- Literature-based discovery (LBD) was performed on a knowledge graph of 33+ million PubMed articles to assess important concepts that related pediatric leukemia to infection. Cross-domain text mining with SemNet 2.0 enabled the comprehensive assessment of the contribution(s) of features not present in the tabular pediatric AML and ALL clinical case study dataset.
- Collectively, the case study successfully formulated initial foundational models that predicted the development of infection in pediatric AML and ALL.

The remainder of the study is organized as follows. Section 2 describes both the general interpretable ML framework developed to analyze rare diseases using small tabular clinical datasets and the application of this framework to predict treatment-related infection in rare pediatric AML and ALL. Section 3 describes the results of the case study, including the stratification of pediatric AML and ALL patients into high infection risk or low infection risk groups, the prediction of the discrete number of infections, the feature importance to ML model prediction(s), and LBD to explore the relative importance of missing features in the dataset using cross-domain text mining. Finally, Section 4 highlights the overall findings of the study.

## 2. Methods

The methods consist of (1) developing a general framework to enable interpretable ML to be applied to small, tabular datasets for the assessment of rare diseases; (2) deploying a real-world applied case study that utilizes the developed general ML framework to predict the development of infection in pediatric AML and ALL.

### 2.1. General Machine Learning Framework for Use of Rare Disease Tabular Clinical Datasets

**Figure 1:**
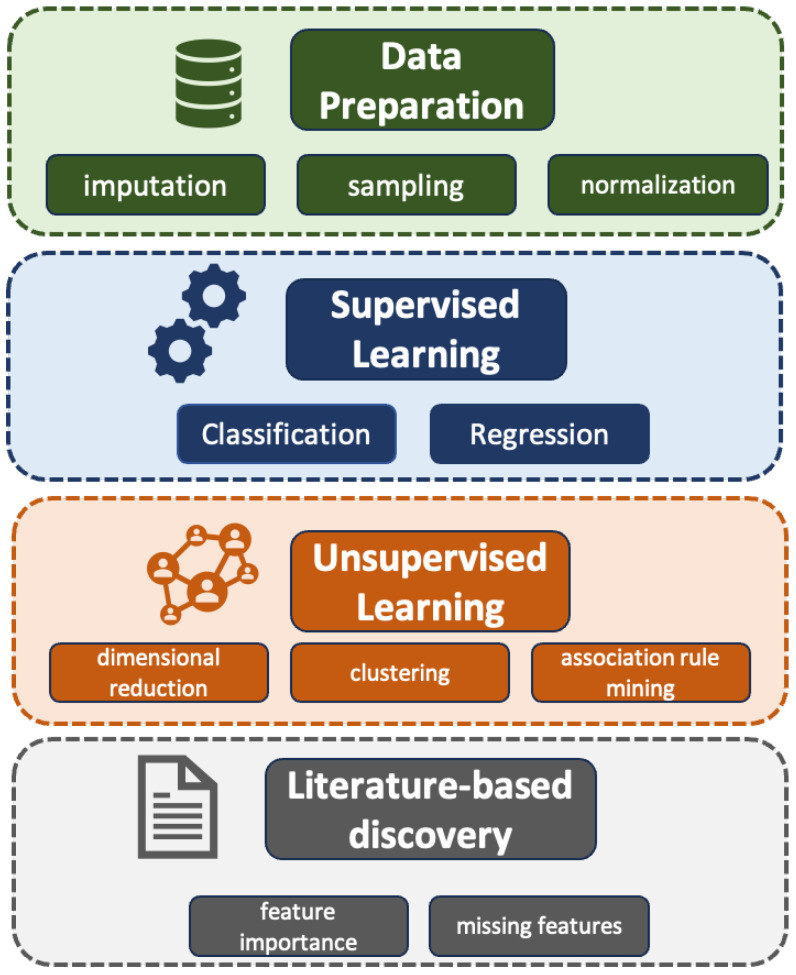
General interpretable machine learning framework adapted for small tabular clinical dataset(s) common to the study of rare disease. The four main modules include data preparation, supervised learning, unsupervised learning, and literature-based discovery. In the presented case study, data preparation was performed first, and the other steps were performed in parallel. While data preparation will always be performed first, the order of the remaining modules could be swapped based on the specific attributes of the dataset, the domain use case, and the explicit research question.

This study developed and assessed a generalizable framework for interpretable ML for small, tabular datasets. As shown in Figure 1, the framework included the following primary modules: data preparation, supervised learning, unsupervised learning, and LBD. Details for each module are described in the following subsections. Briefly, data preparation included preprocessing steps, augmentation, and imputation techniques to optimize the data for input into supervised and unsupervised ML models. Supervised learning, including classification and regression, enables specific predictions using known patients labels and explanatory patient features. Unsupervised learning approaches, such as dimensional reduction, clustering, and association rule mining, elucidate data-driven patterns that best explain outcome variance. Finally, literature-based discovery leverages the vast scientific literature to evaluate the potential value of features that may not be available in a rare disease tabular clinical dataset.

For the presented case study, data preparation was performed first, and the supervised learning, unsupervised learning, and literature-based discovery steps were performed in parallel. While data preparation will always be performed first, the order of the remaining modules in the framework could be swapped based on the specific attributes of the dataset, the domain use case, and the explicit research question. For example, a rare disease dataset with more features than patients may require unsupervised learning to be performed before supervised learning.

### 2.2. Data Preparation

Data preprocessing is vital for any ML pipeline. However, it is especially necessary when dealing with the mixed, variable data types associated with clinical datasets. Raw numerical features are used. Each categorical feature is converted into numerical codes, with a code for each unique feature value. Some models convert these categorical codes into an embedding representation, which is used to train the model.

Missing values must be either imputed or removed. In small datasets, retaining as many samples as possible is critical. The imputation techniques will vary depending on the domain. The risk of over-imputing is that it introduces bias into the data. For instance, if the mean is used as the replacement value, it can shift all missing data towards the mean. K-nearest neighbors (KNN) can also be employed to match missing variables to be similar to patients that have other similar known attributes. The main idea is to utilize imputation techniques that enable the sample size to be retained without overtly altering the signal. Techniques such as one-hot encoding can also be helpful, where unknown values can be assigned a separate attribute indicated by a binary signal.

Rare disease data tend to have small sample sizes and sparsity. Synthetic sampling techniques may be needed to deal with sparsity and to ensure the class balance required for optimal ML. Common approaches to overcome the problem of sparsity and minority classes include (1) oversampling using the Synthetic Minority Oversampling Technique (SMOTE); (2) synthetic patient data generation using the Conditional Tabular Generative Adversarial Network (CTGAN).

### 2.3. Supervised Learning

Supervised learning uses data with known labels to build, train, and test a predictive model. Supervised learning includes either classification or regression. Classification is typically defined as predicting a categorical outcome using a given set of explanatory features. Regression is typically defined as predicting a continuous outcome using a given set of explanatory features.

#### 2.3.1. Model Selection

For supervised learning, different types of models were utilized for each prediction task. The model types were assessed to determine which were best for the majority of prediction tasks with small, tabular datasets.

TabNet was specifically developed to work with tabular data [29]. It uses raw numerical features and maps the categorical features into trainable embeddings without any global normalization. The encoder includes a feature transformer, an attentive transformer, and feature masking. The decoder is composed of feature transformers. The feature selection masks at each step and can show the significance of features in TabNet. It is considered an interpretable neural network.

While tree-based methods are commonly used for classification tasks, they can also be used for regression. The advantage of most tree-based methods is that they are interpretable and follow an intuitive overarching structure that aligns with human reasoning. Common tree-based methods include decision trees and the gradient-boosted ensemble of trees (CatBoost, LightGBM, XGBoost).

In CatBoost, symmetric trees (or balanced trees) refer to the splitting condition being consistent across all nodes at the same depth of the tree. On the other hand, LightGBM and XGBoost result in asymmetric trees where the splitting condition for each node at the same depth can differ. Although both LightGBM and XGBoost produce asymmetric trees, LightGBM grows trees leaf-wise (horizontally), while XGBoost grows them level-wise (vertically). In short, LightGBM grows the tree selectively, resulting in smaller and faster models compared to XGBoost.

#### 2.3.2. Model Evaluation

Models can be evaluated using a split dataset, such as training:validation:test or simply training:test. In either of the aforementioned methods, some data are reserved for training only and some data are reserved for model testing only. Such methods enable the model’s results to be more generalizable and less susceptible to overfitting or noise within the dataset. However, for small datasets with few observations, a cross-validation approach is often preferred. For cross-validation, the data are divided into “folds”. In a five-fold cross-validation design, for instance, 80% of the patients are used to train a model, and the remaining 20% of the patients are used to test the model to obtain the evaluation metrics. This process is repeated five times (once for each fold) to ensure that all patients appear in the test set once. The averaged metrics obtained across the five folds are then reported as the final result. Common model evaluation metrics are described below.

Given the target risk scores, Y, and predicted risk scores, $Y^{\prime }$, over the entire dataset, and the target risk score, Yc, and predicted risk score, $Y^{\prime c}$, for one risk category, *c*, the following metrics are used to compare the efficacy of the models:

- Accuracy $=\frac{\left|Y\cap Y^{\prime }\right|}{N}$;
- Recall for one risk category, $R=\frac{\left|Y^{c}\cap Y^{\prime c}\right|}{\left|Y^{c}\right|}$; the macro-averaged recall is the arithmetic mean of the recall scores obtained for each category;
- Precision for one risk category, $P=\frac{\left|Y^{c}\cap Y^{\prime c}\right|}{\left|Y^{\prime c}\right|}$; the macro-averaged precision is the arithmetic mean of the precision scores obtained for each category;
- F1 score for one risk category $=\frac{2\;*\;P\;*\;R}{P\;+\;R\;}$; the macro-averaged F1 score is the arithmetic mean of the F1 scores obtained for each category;
- AUC-ROC = area under the receiver operating characteristic curve.

#### 2.3.3. Interpretation and Visualization

Interpretability in predictive models is very important for high-stakes scenarios such as healthcare [26,27,30]. Due to their inherent interpretability, decision trees were chosen [31] as the primary method to visualize the model results. Although other tree-based ensemble models are not readily interpretable, there are methods to visualize them. For example, SHapley Additive exPlanations (SHAP) [32,33,34] can quantify the aggregated contribution of each feature and generate the influence of each feature during any inference.

### 2.4. Unsupervised Learning to Assess Relationships

Unsupervised learning does not use pre-labeled data. Rather, the algorithm uses all of the input features to identify novel relationships or patterns that could be of interest. In this work, three different types of unsupervised learning were utilized: (1) dimensionality reduction; (2) association rule mining; (3) unsupervised rank aggregation to identify important relationships from large numbers of biomedical journal articles.

#### 2.4.1. Dimensionality Reduction and Clustering

If the data have many more features than observed samples, dimensionality reduction may be used before supervised learning. Even in small tabular datasets that may not have high dimensionality, unsupervised methods can provide clarity for the stratification of patients or the elucidation of patterns that would not otherwise be obvious.

Principal component analysis (PCA) [35] and t-distributed stochastic neighbor embedding (TSNE) [36] are two common methods of dimensionality reduction.

Clustering methods include simplistic but robust methods like k-means or advanced methods like the density-based clustering algorithm (DBSCAN). DBSCAN may be preferred for high dimensionality, but it requires more skillful hyperparameter optimization.

Often, dimensional reduction and clustering are performed in tandem to improve the results. For example, a common combination is PCA with k-means clustering [37].

#### 2.4.2. Association Rule Mining

Association rule mining (ARM) is a common method used in online shopping carts. In this context, it looks for patterns of purchases among shoppers. When a customer clicks to purchase one item, the algorithm will then direct the website to display related items often purchased together with a message that says “customers like you also bought…”. ARM has also been successfully utilized in clinical data analysis to identify pharmaceutical or disease comorbidities in Alzheimer’s disease [38]. Support values for each association are utilized to assess the relative importance of co-occurring features [38].

#### 2.4.3. Literature-Based Discovery

Literature-based discovery (LBD) can be employed to determine important features for which there may not otherwise be available clinical data. A current state-of-the-art example of LBD software is SemNet 2.0 [39]. SemNet 2.0 identifies relationships between concepts in biomedical text. It constructs a knowledge graph of the concepts (nodes) and the relationships (edges). Unsupervised learning rank aggregation is used to compare metapaths that describe relationships to the user-specified target node(s). A HeteSim similarity score is used to determine the importance of a related source node to the user-specified target node(s) [39]. SemNet 2.0 uses the Unified Medical Language System (UMLS) as its ontology to specify concept types, such as pharmacological substances (PHSU); diseases or syndromes (DSYN) or biologically active substances (BAC); amino acids, peptides, and proteins (AAPP); genes or genomes (GNGM), etc. LBD with SemNet has been very useful for drug repurposing in COVID-19 [40], ascribing mechanisms of resistant hypertension after COVID-19 infection [41], assessing the long-term effects of tyrosine kinase inhibitors in chronic myeloid leukemia [42], and drug repurposing for Parkinson’s disease [43].

### 2.5. Case Study to Predict Infection in Pediatric AML and ALL

The generalized interpretable ML framework described was employed to predict the development of infection in children treated for AML or ALL using a tabular clinical dataset that included data that had previously been collected from children treated at a single institution for ALL or AML as part of another study [44,45]. LBD using the PubMed database of 33+ million articles was employed to identify features not present in the clinical dataset that may otherwise be important to the prediction task.

#### 2.5.1. Patient Cohort

A tabular de-identified clinical dataset was provided by the Aflac Cancer and Blood Disorders Center of Children’s Healthcare of Atlanta under a data use agreement to the Georgia Institute of Technology. The original collection of data for research by the Aflac Cancer and Blood Disorders Center was approved by the Internal Review Board at Children’s Healthcare of Atlanta (Atlanta, GA, USA) under protocol CHOA00000404 on 23 October 2017 and included a patient waiver of consent for analysis due to the retrospective nature of the study. Data were meticulously collected from the electronic health record by (1) trained chart abstractors that followed a detailed chart abstraction guide or (2) automated extraction from an electronic health record with post-extraction curation by a trained epidemiologist. All data were reviewed by a licensed clinician as part of data quality control.

The clinical dataset contained the following information for each patient: patient age at diagnosis; sex; ethnicity; race; type of acute leukemia, including acute myeloid leukemia (AML), T-cell ALL (T-ALL), and B-cell ALL (B-ALL); Down syndrome status (i.e., presence or absence of a Down syndrome diagnosis); white blood cell (WBC) count at the time of initial diagnosis, obtained from a peripheral complete blood count (CBC); minimal residual disease (MRD) status at the end of induction for ALL and end of induction II for AML; the presence or absence of leukemia in the central nervous system at initial diagnosis; the National Cancer Institute (NCI) risk classification group at the time of leukemia diagnosis; chemotherapy information (i.e., course name, number of days elapsed from time of diagnosis until each chemotherapy course began, and specific drug regimen); the presence of infections developed, including the timing and type of infection (i.e., bacterial, viral, fungal, parasitic); and the corresponding chemotherapy course when the infection developed (stage of treatment, including the drug regimen received during the chemotherapy course). The dataset contained 580 patients with ALL (68 T-ALL and 512 B-ALL) and 132 patients with AML. The number of patients in the cohort with/without infection and their NCI risk classification is shown in Table 1.

Missing values in categorical features were imputed by assigning them to a new category. For numerical features, zeros were used to impute missing values. Categorical features are handled in two ways, depending on the model type: (1) categories within each feature are mapped to numerical codes, assigning a unique code to each category; (2) one-hot encoding is used, where each category becomes a separate feature and a ‘1’ indicates the presence of that category.

To address the challenges of sparsity and minority classes in predicting the development of infection, the following approaches were used: (1) oversampling with the Synthetic Minority Oversampling Technique (SMOTE) and (2) generating synthetic patient data via the Conditional Tabular Generative Adversarial Network (CTGAN). Unfortunately, neither of these auxiliary data approaches yielded significant improvements in the results.

#### 2.5.2. Supervised Learning to Predict Infection

Patients were stratified into “high” and “low” infection risk groups. Different models were employed to assess which model type consistently outperformed the others: decision trees and gradient-boosted ensembles of trees, including CatBoost, LightGBM, and XGBoost, as well as the interpretable neural network based on attention, TabNet.

The following models were used for classification: (1) CatBoost [46,47], (2) light gradient boosting machine (LightGBM) [48,49,50], (3) extreme gradient boosting (XGBoost) [51], (4) decision tree [52,53], (5) TabNet [29].

The number of infections is estimated using regression performed with the following models: (1) CatBoost [46,47], (2) LightGBM [48,49,50], (3) XGBoost [51], (4) decision tree [52], (5) ridge regression [52], (6) Gaussian process regressor [52,54], (7) Elastic Net [52,55], (8) TabNet [29].

Five-fold cross-validation was utilized for model evaluation. Average metrics across the five folds are reported. Experiments were conducted using Python 3.9. The model was trained on a server equipped with two Intel Xeon Gold 6136 processors, 384GB RAM, and an NVIDIA Tesla V100 GPU.

#### 2.5.3. Unsupervised Learning in Infection Prediction

The tabular clinical dataset was subjected to PCA and TSNE to reduce its dimensionality into two principal components. Subsequent analyses categorized the reduced features into a high risk and low risk of infection. The visualizations from PCA and TSNE highlighted the separability of the patient cohort based on clinical features that corresponded to a high or low risk of infection.

Association rule mining (ARM) looks for common associations or features that co-occur and can be used to assess the risk of infection development in children being treated for AML or ALL. In particular, ARM was utilized to study the co-occurrence of specific chemotherapy drugs and infection. Relationships of significance between feature value pairs were identified using FP-Growth [56,57].

#### 2.5.4. Literature-Based Discovery to Predict Important Missing Features

SemNet 2.0 was the tool used to identify concepts or features that were not present in the tabular clinical dataset but might be important in predicting the development of infection in pediatric AML or ALL. The terms and corresponding concept unique identifiers (CUIs) for “AML”, “ALL”, “infection”, and “child” were input from the UMLS into SemNet2.0 [39]. SemNet 2.0 mines text from journal articles in the PubMed database to construct a knowledge graph, which highlights relationships between concepts. Unsupervised learning for rank aggregation prioritized the concepts most relevant to the given query. The primary evaluation metric for the SemNet 2.0 model was the HeteSim score. The HeteSim score assessed the relevance of concepts in the graph to the input query. For the present case study, the top ∼1% of the concepts ranked by SemNet 2.0 were manually assessed and compared to the features contained in the tabular clinical dataset. Broad features like “protein” or “hematological disease” were omitted from the SemNet 2.0 results, following the methodology delineated in previous research on cross-domain text mining for chronic myeloid leukemia [42].

## 3. Results and Discussion

The general interpretable ML framework was adopted to examine its efficacy on tabular rare disease clinical data. The main experiments presented in Section 3.1, Section 3.2, Section 3.3, Section 3.4 and Section 3.5 used a tabular clinical dataset composed of 580 pediatric patients with ALL and 132 pediatric patients with AML to stratify patients’ infection risk, predict discrete numbers of infections, and assess which features were most important to infection development. As described in Section 3.6, the cross-domain text mining of 33+ million PubMed articles was performed by employing LBD to identify other features that were not included in the case study dataset but could potentially improve infection risk forecasting in pediatric AML and ALL.

**Figure 2:**
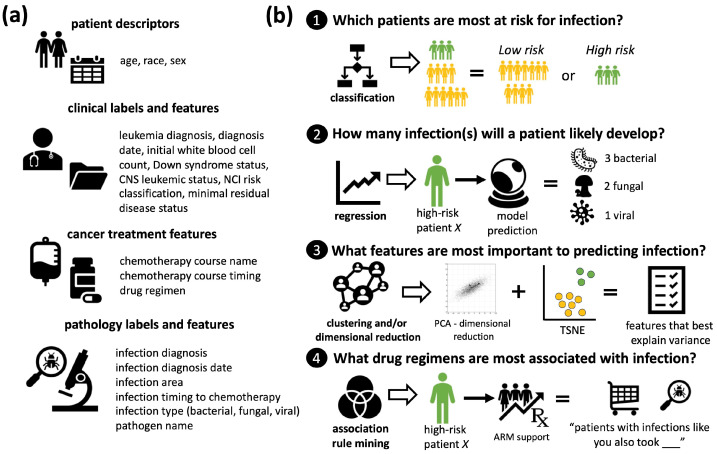
Overview of the clinical case study to predict the development of infection in pediatric AML and ALL utilizing the developed interpretable ML framework for rare disease small tabular clinical datasets. (**a**) Overview of clinical features extracted from the patient records. Features included in the dataset comprised patient descriptors, clinical labels and features, cancer treatment features, and pathology labels and features. (**b**) Application of supervised and unsupervised learning to answer key case study questions.

Figure 2a illustrates the tabular clinical dataset features for children with AML or ALL. Figure 2b illustrates how the supervised learning and unsupervised learning modules were employed in four discrete tasks to assess and predict the development of infection in children with AML or ALL. Task 1 used various classification techniques with data augmentation to stratify the patient infection risk into “high risk” and “low risk”. Task 2 used various regression techniques with data augmentation to predict the discrete number of infections and their types. Task 3 used dimensional reduction to determine which features most explained the infection variance. Task 4 used association rule mining to determine which pediatric AML or ALL drug regimens were most associated with specific infection patterns.

### 3.1. Infection Risk Stratification

It has been established in the literature that patients with AML tend to have more infections than patients with ALL [58]. To provide labels for supervised learning, patients were assigned to high or low infection risk groups based on the presence or absence of a microbiologically diagnosed infection (i.e., presence of a positive microbiological diagnostic test). The type of acute leukemia and other attributes, as detailed in the Methods section, were used as explanatory features to predict the infection risk using supervised classification models.

The evaluation metric results obtained from the supervised classification experiments are displayed in Table 2 for five different model types: CatBoost, LightGBM, XGBoost, decision tree, and TabNet. The CatBoost model had the highest overall accuracy (>79%) and macro precision (0.89) in predicting the infection risk. On the other hand, the TabNet model excelled with the best macro recall (0.68), macro F1 (0.63), and AUC-ROC (0.77).

**Figure 3:**
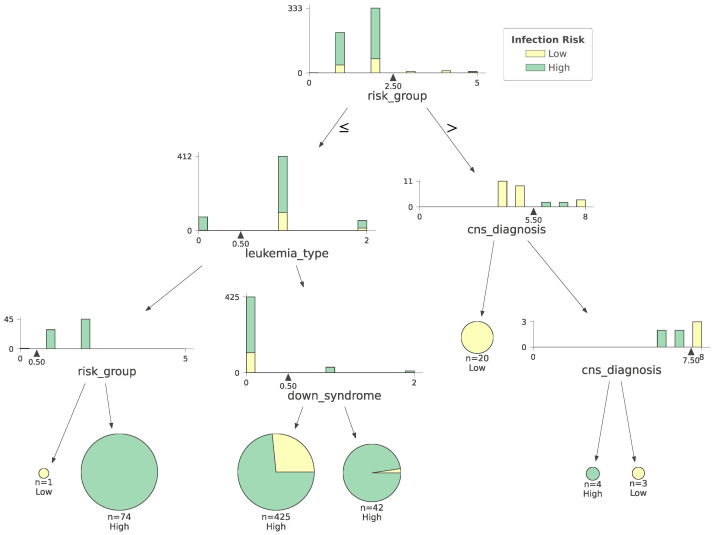
Decision tree for infection risk stratification in pediatric ALL and pediatric AML. Due to space constraints, the tree is pruned to show only the first four decision splits.

A decision tree provides an excellent way to visualize the stratification of patients by infection risk. Each node represents a split in the data. The splits continue until all patients have been classified as either high risk or low risk. A decision tree performing infection risk stratification is shown in Figure 3. For the sake of tractable visualization, the tree shown has been pruned to four levels. The green illustrates the patients with a high risk of infection and the yellow illustrates the patients with a low risk of infection. Beyond the leukemia type, the most predictive attributes shown are the presence/absence of leukemia cells in the central nervous system at the time of initial diagnosis and the presence/absence of Down syndrome at the time of initial diagnosis).

**Figure 4:**
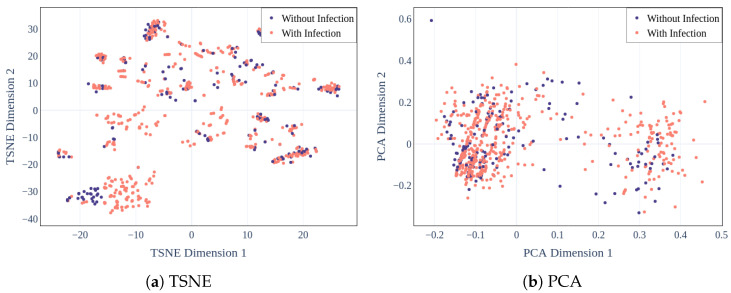
PCA (**a**) TSNE embeddings of stratified ALL and AML infection risk (**b**) PCA visualization of pediatric ALL and pediatric AML infection risk in the reduced two-dimensional feature space.

One way to better interpret the infection prediction model’s results was to assess the separability of the high infection risk and low infection risk classes using unsupervised learning. In particular, dimensionality reduction techniques can visualize the feature space used to build the infection risk stratification model. Principal component analysis (PCA) [35] and t-distributed stochastic neighbor embedding (TSNE) [36] were used to reduce the feature space to two dimensions. The patients with a high infection risk (e.g., had at least one treated infection) are highlighted in red, and those with a low infection risk (e.g., had no treated infection) are shown in blue. The points are visualized in a 2D plot based on the values of the two dimensions in Figure 4.

Thus, according to both PCA (Figure 4b) and TSNE (Figure 4a), the feature space could not fully segregate the two infection risk classes. There is much overlap between the representations of the two infection risk classes. Less than 30% of the infection variance is explained by the first two principal components. Thus, it is difficult to separate the two infection risk classes using solely the clinical features included in the present tabular clinical dataset.

### 3.2. Predicting Discrete Number of Infections

The work above describes the binary classification of AML and ALL patients as high or low infection risk. However, it may be more clinically relevant to forecast the total number of infections that a patient is likely to have over all their courses of chemotherapy. The tabular clinical dataset metrics described in the Methods and in Figure 2 were used as features to make the prediction. For this task, the prediction was split into bacterial and viral infection types. Information was available for fungal and parasitic infections. However, the fungal and parasitic sample sizes were simply too low to reliably implement regression.

An ML implementation of regression with five-fold cross-validation was utilized to predict the quantitative number of infections. Unlike the binary risk classification, the supervised regression model predicts the quantitative number of infections for a given patient. Eight different regression model types were used to explore which regression model methods best predicted the number of infections using a small tabular clinical dataset.

The results obtained from the experiments to predict the total number of bacterial infections in children with AML or ALL are shown in Table 3 for eight regression model types. The best regression model across all evaluation metrics was TabNet. TabNet obtained a mean absolute error (MAE) of 2.15 and a mean squared error (MSE) of 9.28 in predicting the total number of infections. R^2^ (0.095) provides less interpretability in the context of this task, but is shown for completeness. Given the variance and sample size, this result is within the expected limits based on the residual size.

The results for the prediction of the number of viral infections in children with AML or ALL are shown in Table 4 for eight different types of regression models. Here, the best-performing regression model varied based on the evaluation metric. TabNet had the best MAE at 1.26. Ridge regression had the best MSE at 3.43. LightGBM had the best R^2^ (0.062). However, in the context of this task, the MAE is considered the most important and interpretable evaluation metric. As such, TabNet, which had the lowest MAE, is considered the best-performing model to predict the number of viral infections.

**Figure 5:**
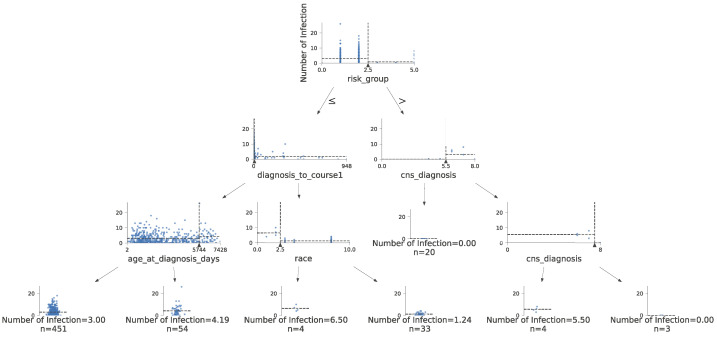
Decision tree to predict the number of infections in children with ALL or AML.

A decision tree regressor provides a way to visually interpret the regression, including which features most contribute to the prediction of the quantitative number of infections. Figure 5 shows a decision tree regressor predicting the number of infections. The tree was pruned to four levels for ease of visualization. Each node shows the distribution values of one feature used to create a split, signified by dotted lines. Notably, the time from leukemia diagnosis to the development of infection and the CNS status at the time of diagnosis are shown as the first attributes used to predict the number of infections.

### 3.3. Anecdotal Comparison of Supervised Model Performance to Clinical Domain Expertise

In short, predicting the binary infection risk classification and/or forecasting the discrete number of expected infections are challenging tasks. The presented models that use standard available clinical features lay an important foundation for the prediction of infection in children with AML and ALL. However, the clinical features that were included in this dataset do not fully explain infection risk. For this reason, the accuracies of the predicted infection risk may seem low compared to predictive models for other disease domains. For example, other clinical domains like sleep staging [28] or epilepsy [59] have shown extremely high accuracy with similar methodologies. However, this is because such models employ much larger sample sizes with many more granular features than were included in the present study’s rare pediatric leukemia dataset. The model prediction accuracies using small tabular datasets for rare disease datasets will likely not approach that of feature-rich datasets with large sample sizes.

Notably, there is no specific infection prediction model baseline from which to compare the performance of the presented regression model(s). Moreover, in these rare pediatric cancers, a quantitative standard infection risk protocol is not presently available as of the time of writing [60]. Most clinicians use neutropenia as the primary feature by which to predict infection [60]. Thus, in short, the presented model outperforms general expectations based on the wide variety of features anecdotally considered by a clinician. Most importantly, the presented infection prediction model(s) provide the first performance baseline(s) by which to compare future prediction models that may be deployed to aid real-time infection prophylaxis treatment decisions.

### 3.4. Exploring Feature Importance in Predicting Infection

Feature importance measures how relevant the feature is to making an accurate prediction. Even when the ML model prediction accuracies may be sub-optimal, the evaluation of feature importance can still provide actionable insights. Here, feature importance was assessed to determine which features generally were most predictive of a pediatric AML or ALL patient’s overall risk of developing an infection(s). The importance ranking of features will naturally vary somewhat across different model types.

**Figure 6:**
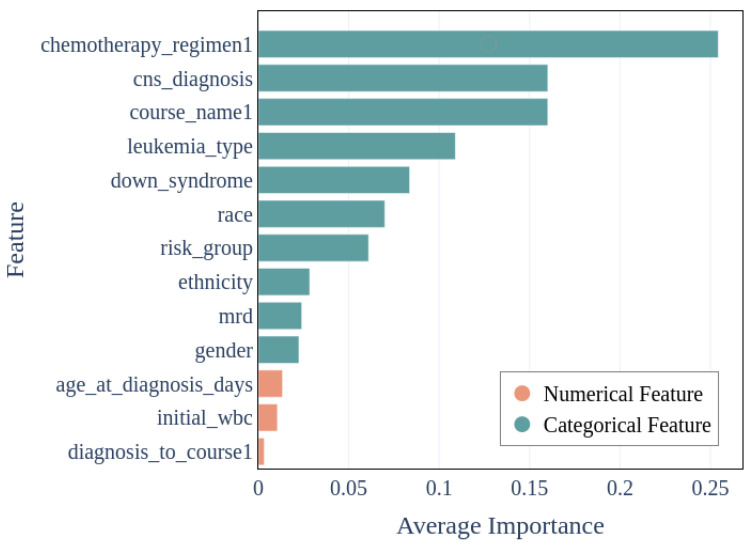
TabNet supervised model feature importance in predicting development of infection in pediatric AML and ALL.

The overall performance evaluation metrics indicate that TabNet was the overall best model in predicting infection in children with AML or ALL. As such, the TabNet results are presented in Figure 6. Note that the most important features in predicting the development of infection with TabNet are the chemotherapy regimen, presence/absence of cancer cells in the central nervous system at the time of diagnosis, chemotherapy course, leukemia type, Down syndrome status, race, and NCI risk classification assigned at the time of initial diagnosis. For completeness, the feature importance for other supervised model types beyond TabNet is shown in Appendix A.

**Figure 7:**
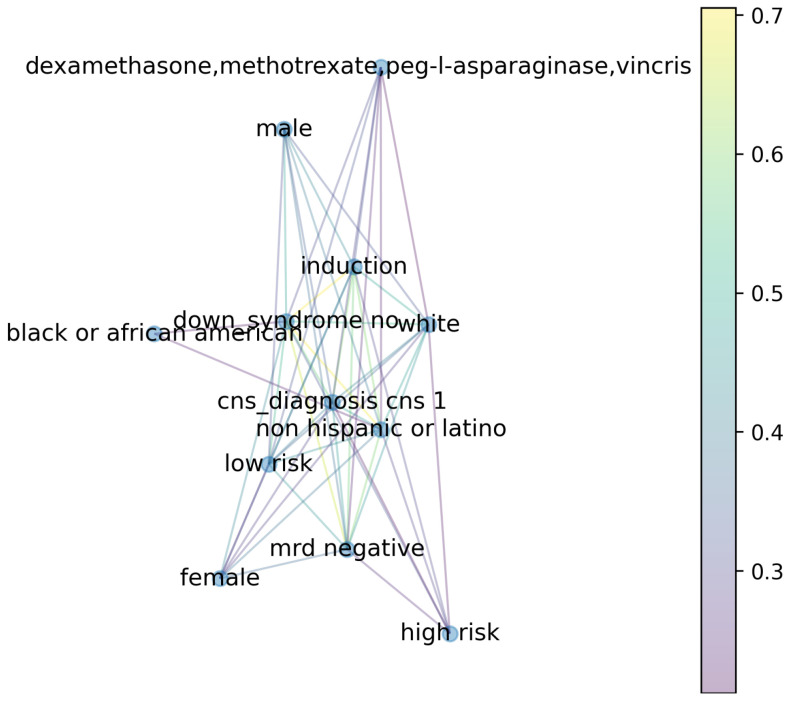
The 50 most significant feature relationships identified with association rule mining (ARM) using FP-Growth. Table A1 shows the support values used to construct the graph.

Another means of exploring the importance of clinical features is through an unsupervised learning method called association rule mining (ARM). ARM is helpful because it does not use pre-labeled data to find patterns. As such, ARM is capable of identifying patterns that may otherwise go unnoticed. For the present work, ARM was used to look for common associations of features that co-occur and could be helpful in assessing the development of infection in pediatric AML and ALL. The most significant relationships between feature value pairs were generated using FP-Growth [56,57]. The support values of the top 50 relationships obtained from the algorithm are shown in Table A1. A graph [61] of the high support relationships is shown in Figure 7, where the color of the graph edges shows the support values. Notably, this figure does not depict the relationships with the chemotherapy treatments, which were also examined separately.

The ARM results support the feature importance rankings from the supervised models, which predominantly indicated the importance of the presence/absence of cancer cells in the CNS at initial diagnosis and the presence/absence of Down syndrome. ARM did identify one other clinical attribute that ranked lower in the supervised models—minimal residual disease (MRD). MRD is the ongoing presence of smaller amounts of cancer cells even after some chemotherapy treatment. Interestingly, ARM illustrated an association between MRD as defined in this clinical dataset (at end of induction for patients with ALL or end of induction II for patients with AML) and the development of more infections. The presence of MRD being associated with higher infection rates is an interesting finding. However, it is crucial to note that the clinical dataset did not include the indication of the MRD status at the time of the infection (i.e., whether the patient had received additional chemotherapy after the initial positive MRD at the end of Induction or Induction II). Nonetheless, the findings of ARM suggest that future research is needed on the association of MRD with the development and treatment of infection(s).

### 3.5. Chemotherapy during Infection

Chemotherapy drug regimens have long been known to cause neutropenia, which increases the risk of infection, requiring intervention [11]. The TabNet importance rankings also show the chemotherapy regimen and course type to be among the most important features in predicting the development of infection in children treated for AML or ALL. Thus, further analysis was performed to better understand which chemotherapy regimens are most associated with the development of infection. Because of the smaller sample size of AML patients, the chemotherapy regimen analysis was limited to the ALL patients.

The clinical dataset contained the chemotherapy regimens that the patient was receiving at the time of infection diagnosis. The top three rows of Table 5 show the drug combinations that appeared most frequently at the time of infection diagnosis in children with ALL. In order to remove anomalous cases of rare drug combinations, the following inclusion criteria were used to identify the top three rows: (1) drug combinations where more than 50 bacterial or viral infections occurred (across all included patients) during the corresponding chemotherapy regimen; (2) a higher number of infections of a particular type occurred during the chemotherapy regimen than the total number of times that it was prescribed (i.e., a higher odds of infection when taking a specific regimen).

Association rule mining (ARM) was also utilized to separately look at the chemotherapy regimes that the patients were taking at the time of infection diagnosis. When methotrexate and vincristine were both included in a chemotherapy regimen, there was the highest support for infection co-occurrence at >0.80. Patients who receive these chemotherapy drugs have been shown to benefit from antimicrobial prophylaxis, although the types of antimicrobial prophylaxis administered may vary [11,12].

### 3.6. Literature-Based Discovery to Identify Missing Features to Predict Infection

The supervised and unsupervised modeling results with tabular clinical data provide a foundation for the prediction of the development of infection in children with acute leukemias. However, the dimensionality reduction analysis made it clear that much of the infection variance was not explained by standard tabular clinical features. Thus, literature-based discovery was performed to better assess which features that were not present in the tabular clinical dataset might improve infection prediction in future work.

**Figure 8:**
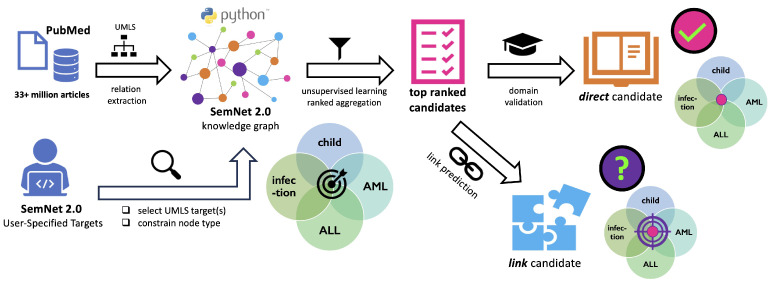
Literature-based discovery using SemNet 2.0 identified and ranked UMLS concepts that were most important to the user-specified UMLS target nodes of “AML”, “ALL”, “child”, “infection”. SemNet 2.0 [39] relationships were extracted from 33+ million journal articles to construct a knowledge graph. The graph was queried to identify and rank the most important concepts, which had relationships that intersected with the target nodes. The top ranked direct candidates were derived from the intersection of highly ranked concepts shared by all 4 target nodes (i.e., area shown in dark pink in the middle of the infection–ALL–AML–child Venn diagram). The top ranked link candidates were relationships that a link prediction algorithm labeled as important using surrounding adjacent literature patterns (i.e., area shown in purple on the infection–ALL–AML–child Venn diagram).

A text-mined knowledge graph based on 33+ million PubMed articles, SemNet 2.0 [39,62], was used to discover relationships between ALL or AML and infection in the clinical literature. SemNet 2.0 has previously proven useful in identifying adverse events for chronic myeloid leukemia [42]. The general framework utilized to employ SemNet 2.0 for the present study is shown in Figure 8. The Unified Medical Language System (UMLS) is utilized within SemNet 2.0 to create the underlying knowledge that connects the graph nodes (e.g., biomedical concepts or keywords). Here, the user-specified target nodes are shown as infection, ALL, AML, child. The full knowledge graph cannot be visualized in a form tractable to the human eye due to the vast number of complex relationships.

SemNet 2.0 was used to find the most important diseases or syndromes (node type DSYN), pharmacological substances (node type PHSU), and biologically active substances (node type BAC) linked to the UMLS nodes of infection, AML, ALL, child. SemNet 2.0 takes in the user-specified UMLS target nodes and then searches and ranks from the knowledge graph the most important related nodes, which are called source nodes. Relatively fewer publications have investigated children with AML or ALL (i.e., pediatric AML or pediatric ALL) compared to adults with AML or ALL. Nonetheless, cross-domain text mining with SemNet 2.0 did identify a few relationships with highly ranked HeteSim scores, which were concepts not present in the case study clinical dataset. Briefly, the SemNet HeteSim score indicates the relative importance of a returned source node in relation to the queried target node(s) [39]. For this analysis, the UMLS target nodes were infection, AML, ALL, and child. The node “child” was included in the search query to better specify pediatric disease, since the UMLS ontology does not otherwise specifically split the disease or syndrome node type (DSYN) into adult and pediatric.

The following were ranked by SemNet 2.0 within the top 1% of returned source nodes: glucose, zinc, iron, growth factors, and lupus. Thus, these nodes are considered to have strong literature-based relationships with infection in AML and/or ALL in children. Notably, the relationships are based on predicted cross-domain patterns across all 33+ million articles and not simply articles on pediatric acute leukemia. The cross-domain text mining approach enables the examination of relationships that may lack direct textual evidence in one field, but the amalgamation of evidence across fields predicts that the relationship is important [42].

The source node “glucose”, a top ranked direct candidate returned by SemNet 2.0, is predominantly tied to hyperglycemia. Hyperglycemia is a relatively common event in pediatric cancer [63,64]. The extent of the role of hyperglycemia in modifying the infection risk is still debated in pediatric cancer. However, there is some clinical cohort study evidence to indicate that hyperglycemia, including transient hyperglycemia, is associated with increased neutropenia and overall poorer prognoses [65,66]. Treatment-induced diabetes [67] is another adverse event in pediatric ALL that has also been shown to increase the risk of developing an infection.

SemNet 2.0 also returned micronutrients like zinc and iron as highly ranked direct candidates associated with infection, acute leukemia, and children. A recent study found that the supplementation of zinc significantly decreased infection rates in children and adolescents undergoing chemotherapy for ALL [68]. Likewise, a recent study found that iron influences the progression of acute leukemia and the occurrence of infection during chemotherapy [69].

The source node “growth factors”, returned by SemNet 2.0, has been utilized to combat the effects of neutropenia in children with AML or ALL. For example, granulocyte colony stimulating factor has been given for AML [70] but remains controversial due to limited efficacy in reducing neutropenia and an increased risk of AML relapse. Evidence from ALL trials with hematopoietic growth factors is mixed; some studies suggest a reduction in severe infections by myeloid growth factors, whereas others report no effect [71]. Growth factors, like VEGF-A, have also been tied to CNS invasion in pediatric leukemia [72].

The only source node within the top 1% of the SemNet 2.0 returns related to a non-hematological disease or syndrome was “lupus” or systemic lupus erythematosus (SLE). Studies have shown a higher incidence of leukemia, especially ALL, among adult lupus patients [73]. Interestingly, the genetic underpinnings of lupus, including genome-wide association studies (GWAS), have been found to be quite similar to lymphoma [74]. Likewise, in the limited clinical research examining children with SLE, it has been shown that there is an increased association of malignancy, and especially hematologic malignancy [75], in children with SLE.

As shown in Figure 8, direct candidates are top ranked concepts derived from explicit, existing literature relationships from the intersecting target nodes. In contrast, link candidates are top ranked concepts derived from a link prediction algorithm that examined adjacent literature patterns. Lupus was a top ranked direct candidate when considering only the AML–ALL–infection intersection but was a top ranked link candidate when also including the child node. The nuanced difference in the top ranked candidate type is indicative of the lesser volume of literature data on children that have both an acute leukemia and SLE. Link prediction is valuable when there is either a relatively new node or a node with a smaller number of data sources. For example, link prediction was able to use patterns from other prior, historical SARS viruses in the literature to predict potential repurposed drugs for the emergent SARS-CoV-2, which had much less published data available to include in the knowledge graph at the time of SemNet analysis [40]. In the present study, link prediction was helpful because of the relatively smaller number of data sources connected to the child node with AML, ALL, and infection.

Other SemNet 2.0 results in the top 1% of returned source nodes included features that were already in the tabular clinical dataset and corresponding ML models, such as Down syndrome, central nervous system infiltration, and age. A recent review on Down syndrome and leukemia investigated and compared trends in treatment-related morality and relapse [76]. Central nervous system infiltration has long been considered an important factor in describing potential pediatric leukemia prognoses. Recent work found that the co-detection of the growth factor VEGF-A and microRNA-181a may indicate central nervous system involvement in pediatric leukemia [72]. Finally, there is a plethora of evidence that shows older age, namely >10 years of age at diagnosis, to be associated with more negative outcomes in pediatric acute leukemias [77]. The collective selection of these high-ranking concepts from the literature-based discovery algorithm, SemNet 2.0, provides further confidence in the feature importance results of the presented ML framework using tabular clinical data.

### 3.7. Limitations and Future Directions

The primary limitations of this case study were the small sample sizes and the limited number of data elements included in the tabular clinical dataset. These are inherent real-world limitations ascribed to most rare disease tabular clinical datasets. The presence of higher-dimensional or higher-resolution temporal features might better explain the variance in the infection prediction signal. Nonetheless, the developed interpretable ML framework for rare disease small tabular datasets provided actionable insights using standardly available clinical features. Additional methodological assessments and future work include the following:

- The pros and cons of data augmentation—Some researchers in the healthcare domain may not consider imputing data or performing synthetic oversampling or undersampling due to the fear of bias. While the imputation and oversampling methods utilized here did not result in large performance gains, the authors contend that both steps are critical to the success of ML in most small tabular datasets. Data augmentation has been shown to be pivotal to improving performance in other clinical applications like epileptic monitoring [59]. In epilepsy sensor data, the overall number of data points labeled as a seizure is often exceedingly small, which results in a very imbalanced dataset. Data augmentation successfully handled the challenges of small sample size and class imbalance [59].
- The trade-off of accuracy and interpretability—The work presented here focused on interpretable ML methods, which are also sometimes referred to as explainable AI [26]. Interpretable and/or explainable methods make it easier to see why the model is making a particular prediction. It is possible that less interpretable black box methods might make better predictions [28]. However, black box methods that employ large neural networks need very large sample sizes, often more than 10,000 patients [59,78]. There is often a trade-off between accuracy and interpretability [28], but advances in ML are narrowing this gap [59,78].
- Use of probabilistic generative models—Another possibility for future work to apply ML to small tabular datasets for rare disease is probabilistic generative models. Probabilistic generative models, such as the recent scaled event-based model (sEBM), can use multimodal, cross-sectional data to stratify patient populations and/or disease progression [79]. These advances enable temporal or longitudinal modeling in the absence of large-sample-size longitudinal data.
- Use of transfer learning—Transfer learning, which applies knowledge gained from a larger distribution or dataset to a smaller one, could be added as a module to the proposed general framework for specific research use cases [80] However, in general, transfer learning would not be as generally suited to all rare disease, particularly heterogeneous rare diseases, because their sample distributions may not be well represented by the larger aggregate or average model distribution.
- Use of large language models—Large language models like ChatGPT may enable the aggregation and extraction of multiple published rare disease datasets in order to increase the available sample sizes for standard collected features [81]. While large language models excel in producing tabular data from unstructured data, most are currently not specifically suited for the generation of predictions using small-sample-size tabular data.

## 4. Conclusions

This study developed a general interpretable ML framework to enable actionable insights from small rare disease tabular datasets, which traditionally have not been amenable to ML. The general framework combined data processing, supervised learning, unsupervised learning, and LBD to maximize the derived insights. Each integrated ML module and method enabled a different perspective on the data. The general framework was used to describe infection development in children with AML or ALL using a small, tabular clinical dataset. The specific case study conclusions are as follows.

- The best supervised learning model for infection risk stratification for children with AML or ALL resulted in an accuracy of 79%. At the time of writing, there were no known pediatric AML or ALL infection prediction models with which to compare the presented models’ performance. As such, the presented model(s) lay a critical foundation and performance baseline for future, real-time clinical prediction models to optimize personalized infection prophylaxis treatment decisions.
- The features that most explained the development of infection were the type of chemotherapy regimen, the presence of cancer cells in the CNS at initial diagnosis, the chemotherapy course, the leukemia type, the Down syndrome status at diagnosis, race, and the NCI risk classification.
- ML enabled the cross-domain text mining of over 33 million PubMed articles, which indicated that future models should consider glucose, iron, zinc, growth factors, and lupus status as additional features for consideration when evaluating the development of infection in pediatric AML and ALL.

## Figures and Tables

**Table 1:** Infection data for the patient cohort as a function of leukemia type. T-ALL: T-cell acute lymphoblastic leukemia. B-ALL: B-cell acute lymphoblastic leukemia. AML: acute myeloid leukemia. With Infection: Patients who developed at least one infection during the chemotherapy courses included in the cohort. Without Infection: Patients who did not develop any infection during the chemotherapy courses included in the cohort. For T-ALL and B-ALL: standard and intermediate risk are combined into NCI Standard Risk.

Leukemia Type	With Infection	Without Infection	NCI High Risk	NCI Standard Risk
T-ALL	50	18	24	43
B-ALL	382	130	201	311
AML	101	31	53	67

**Table 2:** Results of the proposed models predicting the development of infection in children treated for ALL or AML using interpretable methods. This table presents the average metrics obtained from 5-fold cross-validation. The best results for each column are highlighted using bold and underline typeface.

Model	Accuracy (%)	Macro Precision	Macro Recall	Macro F1	AUC-ROC
CatBoost	**79.1**	**0.89**	0.58	0.58	0.69
LightGBM	77.1	0.71	0.59	0.59	0.69
XGBoost	77.8	0.74	0.59	0.60	0.70
Decision Tree	78.9	0.87	0.58	0.58	0.69
TabNet	67.4	0.64	**0.68**	**0.63**	**0.77**

**Table 3:** Results for supervised regression with 5-fold cross-validation to predict the number of bacterial infections in children with ALL or AML. The best result for each evaluation metric is highlighted using bold and underline typeface. The TabNet regression model had the best performance across all evaluation metrics.

Model	Mean Absolute Error	Mean Squared Error	R^2^
CatBoost	2.29	10.08	0.011
LightGBM	2.26	10.11	0.012
XGBoost	2.30	9.84	0.037
Ridge Regression	2.30	9.82	0.037
Decision Tree	2.30	10.19	0.001
Gaussian Process Regressor	2.37	10.20	0.001
Elastic Net	2.36	10.16	0.004
TabNet	**2.15**	**9.28**	**0.095**

**Table 4:** Results for supervised regression with 5-fold cross-validation to predict the number of viral infections in children with ALL or AML. The best result for each evaluation metric is highlighted using bold and underline typeface.

Model	Mean Absolute Error	Mean Squared Error	R^2^
CatBoost	1.32	3.55	0.037
LightGBM	1.29	3.46	**0.062**
XGBoost	1.33	3.54	0.041
Ridge	1.33	**3.43**	0.061
Decision Tree	1.31	3.57	0.038
Gaussian Process Regressor	1.32	3.47	0.055
Elastic Net	1.32	3.46	0.061
TabNet	**1.26**	3.57	0.027

**Table 5:** Most recent chemotherapy regimens at the time of infection detection in children treated for ALL. Only confirmed infections are included. The first three rows show chemotherapy regimens during which an unusually high number of bacterial or viral infections occurred. These three rows represent the only chemotherapy regimens that satisfy the following two conditions: (1) more than 50 bacterial or viral infections occur during the corresponding chemotherapy regimen; (2) the number of bacterial or viral infections is approximately equal to or exceeds the number of times that the chemotherapy regimen was prescribed.

Chemotherapy Regimen	Prescriptions	Bacterial Infection	Viral Infection
daunorubicin; dexamethasone; methotrexate; peg-l-asparaginase; vincristine	54	59	8
6-mercaptopurine (6-mp); dexamethasone; methotrexate; vincristine	198	87	315
6-mercaptopurine (6-mp); methotrexate; prednisone; vincristine	96	36	87
6-mercaptopurine (6-mp); methotrexate; vincristine	297	39	54
daunorubicin; methotrexate; peg-l-asparaginase; prednisone; vincristine	84	35	12
methotrexate; vincristine	206	14	15

**Figure A1:**
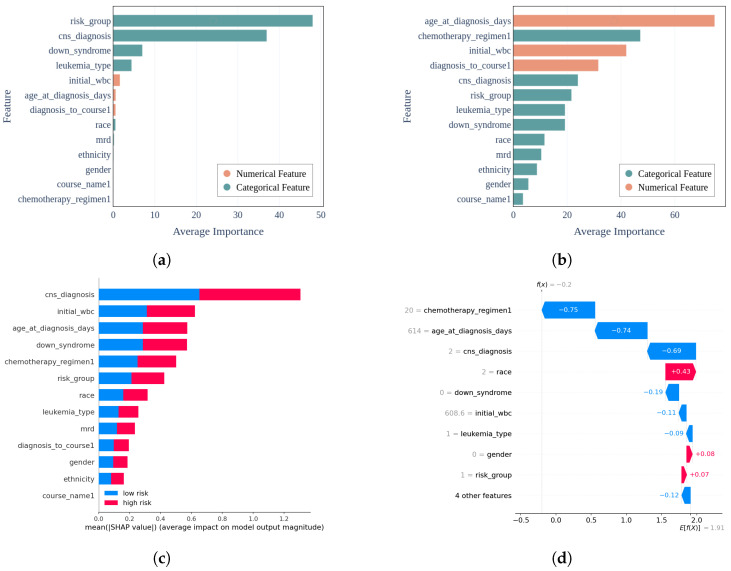
Feature importance for infection risk stratification of pediatric ALL and pediatric AML for additional supervised models. (**a**) CatBoost: feature importance. (**b**) XGBoost: feature importance. (**c**) LightGBM: feature importance for each class using SHAP. (**d**) LightGBM: effect of features for one subject using SHAP.

## Data Availability

The clinical case study dataset is a privately maintained database by the Aflac Cancer Center. Inquiries should be made to T.P.M. The SemNet 2.0 software is an open source code that can be found on GitHub https://github.com/pathology-dynamics/semnet-2 (accessed on 18 January 2023).

## Appendix A. Infection Risk

Figure A1 displays the significance of features in the gradient-boosted trees for the prediction of infection risk. Similarly, Figure A1a,b and Figure 6 detail the feature importance for CatBoost, XGBoost, and TabNet, respectively, in predicting infection risk. Different features exhibit importance across the various models. This underscores the significance of feature representation, especially when building predictive models from tabular clinical data. Figure A1c highlights the influence of each feature in determining high and low infection risk, with each feature having a consistent impact on the class prediction. Figure A1d illustrates the significance of the top features and their corresponding raw values when predicting for a specific patient. The encoded categorical variables are represented in gray on the y-axis, with the numerical variables’ raw values also presented. The final row demonstrates the cumulative effect of four features.

**Table A1:** Top 50 most significant relationships between feature values used for infection risk stratification of acute pediatric lymphoblastic and myeloid leukemia according to FP-Growth.

Rank	Feature 1	Feature 2	Support
1	down_syndrome no	induction	0.705
2	down_syndrome no	non hispanic or latino	0.697
3	down_syndrome no	mrd negative	0.664
4	induction	non hispanic or latino	0.597
5	cns_diagnosis cns 1	induction	0.597
6	mrd negative	non hispanic or latino	0.591
7	induction	mrd negative	0.579
8	cns_diagnosis cns 1	down_syndrome no	0.573
9	down_syndrome no	white	0.535
10	down_syndrome no	low risk	0.524
11	induction	white	0.485
12	non hispanic or latino	white	0.479
13	down_syndrome no	male	0.476
14	induction	low risk	0.475
15	cns_diagnosis cns 1	non hispanic or latino	0.475
16	cns_diagnosis cns 1	mrd negative	0.463
17	low risk	mrd negative	0.451
18	mrd negative	white	0.445
19	low risk	non hispanic or latino	0.437
20	induction	male	0.426
21	down_syndrome no	female	0.423
22	cns_diagnosis cns 1	white	0.420
23	cns_diagnosis cns 1	low risk	0.412
24	male	non hispanic or latino	0.397
25	female	non hispanic or latino	0.381
26	male	mrd negative	0.378
27	female	mrd negative	0.362
28	low risk	white	0.358
29	female	induction	0.351
30	cns_diagnosis cns 1	male	0.348
31	down_syndrome no	high risk	0.333
32	male	white	0.322
33	dexamethasone,methotrexate, peg-l-asparaginase,vincristine	induction	0.320
34	dexamethasone,methotrexate, peg-l-asparaginase,vincristine	low risk	0.320
35	low risk	male	0.313
36	dexamethasone,methotrexate, peg-l-asparaginase,vincristine	down_syndrome no	0.306
37	high risk	induction	0.299
38	high risk	non hispanic or latino	0.296
39	cns_diagnosis cns 1	dexamethasone,methotrexate, peg-l-asparaginase,vincristine	0.292
40	cns_diagnosis cns 1	female	0.282
41	female	white	0.282
42	female	low risk	0.265
43	high risk	mrd negative	0.256
44	dexamethasone,methotrexate, peg-l-asparaginase,vincristine	mrd negative	0.243
45	dexamethasone,methotrexate, peg-l-asparaginase,vincristine	non hispanic or latino	0.237
46	black or african american	non hispanic or latino	0.235
47	black or african american	down_syndrome no	0.226
48	high risk	white	0.226
49	cns_diagnosis cns 1	high risk	0.219
50	dexamethasone,methotrexate, peg-l-asparaginase,vincristine	white	0.212
